# The specialized pediatric palliative care service in Italy: how is it working? Results of the nationwide PalliPed study

**DOI:** 10.1186/s13052-024-01604-1

**Published:** 2024-03-19

**Authors:** Franca Benini, Anna Mercante, Sara Di Nunzio, Simonetta Papa, The PalliPed Working Group, Caterina Agosto, Beatrice Albanesi, Sergio Amarri, Irene Avagnina, Elisa Barbugian, Rosaria Basile, Ornella Bellagamba, Francesca Bellini, Cristina Beltrami, Elisabetta Bignamini, Marco Bolognani, Marta Campagna, Caterina Carraro, Gaetano Catalano, Igor Catalano, Loredana Celentano, Maria Grazia De Marinis, Valentina De Tommasi, Lucia De Zen, Antuan Divisic, Anna Dolcini, Alessandra Fasson, Francesca Franchi, Grazia Ghiraldo, Luca Giacomelli, Enrica Grigolon, Antonio Iadelica, Pierina Lazzarin, Irene Maghini, Luca Manfredini, Anna Marinetto, Elisa Mazzoni, Elisa Michelotto, Roberta Mirone, Grazia Molinaro, Paola Moliterni, Nicoletta Moro, Rocco Orofino, Giuseppina Paone, Federico Pellegatta, Carlo Peruselli, Ulrike Veronika Piccolruaz, Marina Raspi, Barbara Roverato, Michele Salata, Anna Santini, Barbara Schiavon, Silvia Soffritti, Valentina Taucar, Marlis Thaler, Assunta Tornesello, Francesca Uez, Silvia Vaccher, Cesare Vezzoli, Anna Zanin, Stefania Ziggiotto

**Affiliations:** 1 Pediatric Palliative Care, Pain Service, Department of Women’s and Children’s Health, University of Padua, Padua, Italy; 2 Department of Biomedical and Neuromotor Sciences, University of Bologna, Bologna, Italy; 3 Polistudium SRL, Milan, Italy

**Keywords:** Pediatric palliative care, PPC, Life-limiting conditions, Life-threatening conditions, Terminal illness, Network, Organization

## Abstract

**Background:**

Accurate estimation of the specialized pediatric palliative care (PPC) burden and the definition of the extent and quality of PPC service in Italy represent urgent needs to enable the proper allocation of PPC resources and the definition of prevention and educational plans. The PalliPed project aimed to provide the first comprehensive assessment of the characteristics of Italian patients requiring PPC, the quality and extent of regional PPC networks/facilities, and the number of dedicated resources. In this paper, we present the results of the second part of the project, regarding the implementation and quality of PPC services in Italy.

**Methods:**

The PalliPed study had an observational cross-sectional design. All Italian specialized PPC centers/facilities were invited to participate in the project and complete a survey on the characteristics of PPC centers/facilities in different care settings, reporting data as of 24 October 2022. Data were collected online.

**Results:**

19 PPC specialized centers/facilities from 12 Italian regions and two autonomous provinces responded to the survey. Among them, 11 are regional referral centers. Seven Italian regions out of 20 reported no PPC centers/facilities, mainly in central-southern Italy. Less than half (45%) of the regional referral centers cover the entire regional territory, and three offer 24/7 service. Ten centers have a dedicated team. Half of the eight non-referral centers offer 24/7 service and have a dedicated team. A total of 1,092 patients were reported by 18 centers as of 24 October 2022. Over the years, an increasing number of patients has been reported, rising from 1,202 (2019) to 1,544 (2021). The dedicated staff is inadequate, and most healthcare providers are not recognized at an institutional level. A shortage of ‘young’ staff and a lack of specific training was reported, particularly among nurses (77% had no training in PPC).

**Conclusions:**

The results obtained show how training, information, and research interventions are still necessary for the reorganization of the available resources and definition of proper strategies to respond dynamically to the new emerging needs of these populations. At the same time, our study represents a first step in defining a national registry of PPC models, useful for monitoring evolutions, and critical issues and planning any new or corrective strategy.

**Supplementary Information:**

The online version contains supplementary material available at 10.1186/s13052-024-01604-1.

## Introduction

Pediatric palliative care (PPC) aims to address all the physical and psychological needs of children affected by life-limiting and life-threatening conditions and their families and to improve their quality of life by establishing a solid network of assistance and care [1, 2]. Children needing PPC may have a wide range of conditions, including life limiting and life-threatening diseases, malignant tumors, neurodegenerative and neurological disorders, premature birth, and congenital abnormalities [3, 4]. Importantly, PPC must not be limited to the terminal or end-of-life phase: a PPC service should be established once the “incurable” or life-threatening illness is diagnosed and should be continued regardless of whether a child receives treatment for the disease [1]. Indeed, unlike the palliative care setting for adults, children who need PPC are predominantly affected by rare, genetic, neuromuscular, post-anoxic, and more rarely oncological diseases. This determines the need for a PPC pathway peculiar in terms of duration and type of needs and, therefore, in terms of the type of assistance needed. In this context, the goal of treatment is no longer aimed at healing, but at the ‘maximum health’ and ‘quality of life’ possible.

In recent years, the prevalence of children with incurable diseases and/or severe disabilities has increased markedly. Medical and technological advances have reduced the mortality associated with these diseases, prolonging the survival of patients and thus generating new care needs that PPC must address [1]. Indeed, thanks to these advances, these patients often live for a long time with multiple organ failures, with cognitive and/or neuromotor problems, whose lives often depend on the ‘machines’ and with a real and daily risk of aggravation and death. This leads to the onset of complex needs and unique care requirements for longer periods, often integrated, multi-specialist and inter-institutional, overall creating a new category of patients in the clinical practice of PPC. Hence, PPC service can last for years and may change according to the disease progression, the child’s development, and the evolution of symptoms, encompassing several dimensions (social, relational, spiritual, ethical) and requiring integrated care offerings.

Therefore, the global need for PPC worldwide is high: according to WHO, it is supposed to involve approximately 21 million patients [3]. A recent estimate indicates that 20,540–32,864 children in Italy require PPC, accounting for 34–54 children/100,000 inhabitants, of whom 18/100,000 require a specialized PPC service, defined as a dedicated setting including an interdisciplinary team of experts in PPC [1, 5].

Given the extension and complexity of patients requiring specialized PPC, a multidisciplinary network of PPC specialists must be established to address the family needs and make use of available community resources, with the widest possible diffusion on the national territory and a 24/7-hour service [6]. Consequently, evaluation of specialized PPC service quality is imperative for a better organization, which in turn translates into improved outcomes for patients and families [7]. This urgency is further strengthened by the Italian Law 38/2010, which claims a specialized response to the needs of children on PPC and their families, both clinically and organizationally, through the implementation of a network of PPC-specific services, acting regionally under the coordination of a single referral center [8]. According to this model, a specialized and interdisciplinary team afferent to the referral center is responsible for coordinating and supporting 24/7 the care activity of the entire network, taking charge of children and their families in all care settings (pediatric hospice and possibly hospital). However, information on its actual implementation in Italy is scant, and this gap hampers the organization of PPC by Italian policymakers.

Recently, we have completed the nationwide PalliPed project, which aimed to collect information on the PPC burden in Italy, assess the quality of regional specialized PPC networks/facilities, and characterize this model of care. The results concerning the Italian PPC burden have already been published elsewhere [9]. Briefly, 867 out of 1,029 reported patients were described, providing the first comprehensive demographic and clinical database of patients requiring specialized PPC in Italy; reported data suggested the lack of adequate PPC service extension in Italy, according to the available estimate of PPC needs (at the time of the survey, the need for PPC in Italy was covered by only 15%). Most patients were between 6 and 16 years old (45%), and patients < 1 month were six (0.8%), a lower percentage than the reported rate of deaths occurring before 28 days of age, suggesting the lack of a prompt referral to PPC by pediatricians or territorial services. At the same time, the need for more family support emerged, particularly to support the mothers most affected by this situation at work and social levels [9]. In addition, an improvement of healthcare providers’ communication skills seemed necessary, to ensure greater involvement of patients and families in care decisions [9]. The need for improved family support measures was also reported [9].

Here, we present the results concerning the implementation and quality of specialized PPC services in Italy to help policymakers better design the PPC network and define support measures at the Italian level.

## Methods

### Project overview

A detailed description of the PalliPed project, coordinated by The Pediatric Pain and Palliative Care Service of Padua University, has already been provided elsewhere [9]. All Italian centers/facilities providing specialized PPC were invited to participate. Centers that provide standard and basic PC activities in the pediatric departments, without providing specialized PPC, were not considered. To reach all the PPC centers, all regional institutions were involved and all regional resolutions and regulations in this regard, which are required by law, were evaluated. Participating centers were asked to complete a two-step self-reporting survey considering data as of 24 October 2022. The first step consisted in collecting patients’ and families’ data, the second on PPC centers/facilities in different care settings. Here, we present the result of the second part of the survey.

The study was conducted in accordance with the Declaration of Helsinki, and the protocol was approved by the Ethics Committees of all participating centers. All the participants ≥ 18 years or legal guardians for younger children gave their consent to the use of medical records for research purposes.

### PPC service survey

The specialized PPC service survey consisted of 28 questions, categorized into four areas: (i) general information, such as the region in which the service is provided, care settings, and number of patients followed as of 24 October 2022 (six items); (ii) type and characteristics of service, including territorial coverage, 24/7 availability, presence of a dedicated team, number of beds (14 items); (iii) number of patients assisted in the period 2019–2021 (three items); (iv) number of healthcare providers working in or with the facility, expressed as the total number and full-time equivalent (FTE; one FTE is intended as a person working full time, e.g., two part-time workers correspond to one FTE) and their education/training (five items). The survey is available as a supplementary material (Appendix I).

### Statistical analysis

All data were analyzed using descriptive statistics. The SPSS vs. 2214 software was used for data analysis.

## Results

### General information

In total, 19 specialized PPC centers/facilities from 12 Italian regions (Basilicata, Campania, Emilia-Romagna, Friuli, Lazio, Liguria, Lombardy, Piedmont, Puglia, Sicily, Tuscany, Veneto) and two autonomous provinces (Trento and South Tyrol) participated in the survey (Table 1). The remaining seven Italian regions have no specialized PPC centers/facilities.

**Table 1:** Regions of the 19 participating centers

Regions	Centers/facilities, *n* (%)
Basilicata	1 (5.3)
Campania	1 (5.3)
Emilia Romagna	2 (10.5)
Friuli	2 (10.5)
Lazio	1 (5.3)
Liguria	1 (5.3)
Lombardy	2 (10.5)
Piedmont	1 (5.3)
The autonomous province of Trento	1 (5.3)
Puglia	2 (10.5)
Sicily	2 (10.5)
Tuscany	1 (5.3)
The autonomous province of South Tyrol	1 (5.3)
Veneto	1 (5.3)

**Fig. 1:**
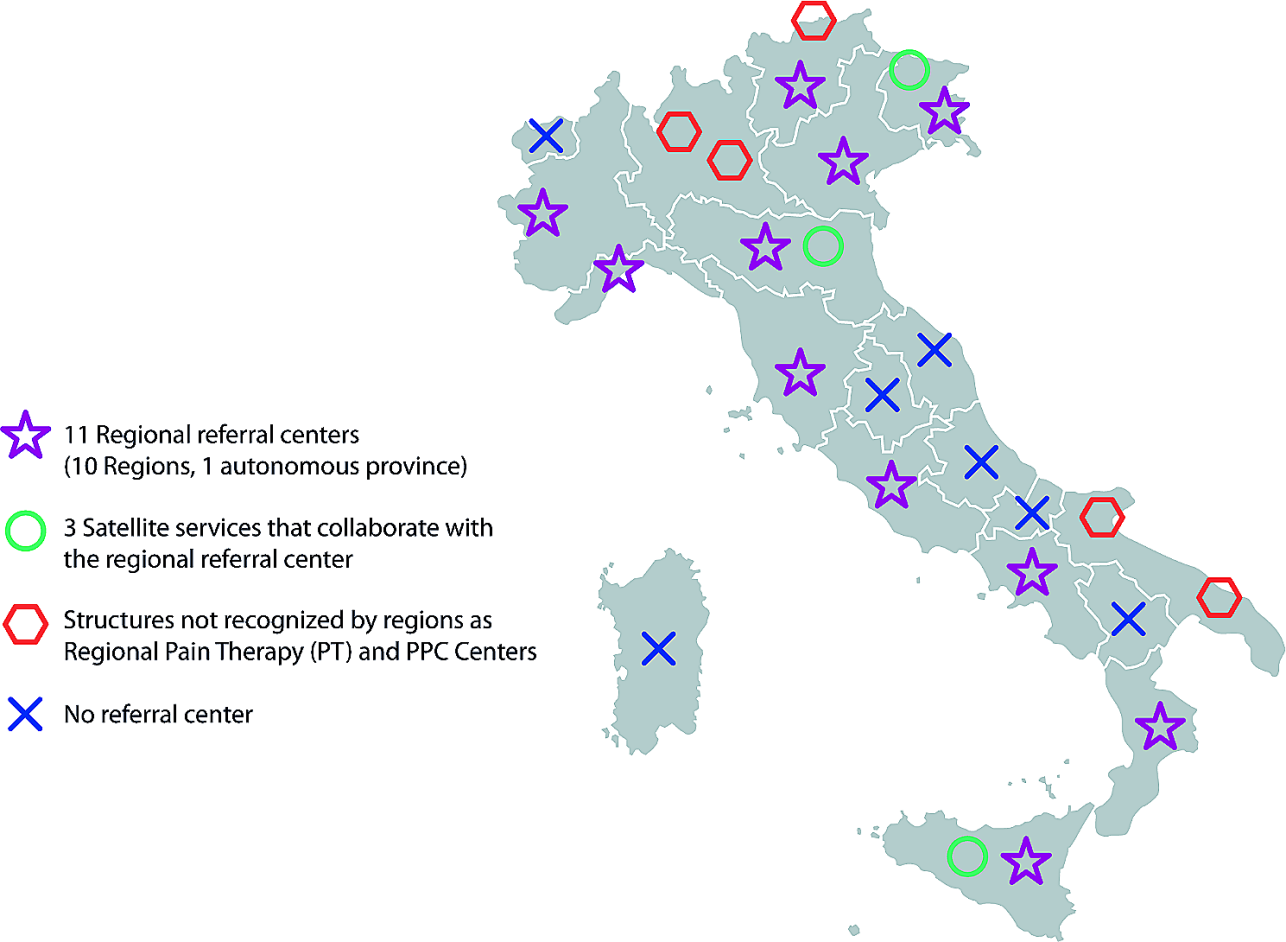
Distribution of the 19 PPC Centers participating in the PalliPed project

Of the 19 PPC centers, 14 (74%) are within the national healthcare system (NHS), four (21%) are accredited private entities, and one (5%) is a non-profit organization. Eleven (58%) are regional referral centers: Basilicata, Campania, Emilia Romagna, Friuli, Lazio, Liguria, Piedmont, Sicily, Tuscany, Veneto, the autonomous province of Trento (Fig. 1; please see supplementary material for definitions); Emilia Romagna, Friuli, and Sicily have satellite services that collaborate with the regional referral center (Fig. 1); Lombardy, Puglia and the autonomous province of South Tyrol have structures not recognized by the regions as regional pain therapy (PT) and PPC centers, of which one has a pediatric hospice (Fig. 1; please see supplementary material for definitions). There is no referral center in seven (26%) regions (Fig. 1).

Reported care settings are summarized in Table 2.

**Table 2:** Care settings

Settings	*n* (%)
Pediatric hospice	8 (42.1)
Home	13 (68.4)
Hospital outpatient clinic	14 (73.7)
Hospital	13 (68.4)
Others	5 (26.3)

Overall, 1,092 patients with PPC needs were reported as of 24 October 2022; the median number of followed patients per center was 46 (range 2–243). Seven centers (39%) were following < 30 patients, seven (39%) were following 20–60 patients, and four (22%) were following > 60 patients.

### Type and characteristics of service

Among the 11 regional referral centers, five (45%) cover the entire regional territory (Veneto, Basilicata, Liguria, Trentino, Friuli), and three (27%) offer 24/7 service (Basilicata, Liguria, Veneto) (Table 3). In Emilia Romagna, Friuli, and the autonomous region of Trento, the 24/7 service is integrated with primary and/or palliative care for adult patients. Five regions have centralized their work on pediatric hospices or hospitals, with limited networking activity. Ten referral centers have a dedicated team (all centers excluding Piedmont).

**Table 3:** Continuity of care

	Referral centers (*n* = 11), n (%)	Other centers (*n* = 8), n (%)
24/7	3 (27.2)	4 (50.0)
8–20 h	5 (45.4)	1 (12.5)
8–16 h	1 (9.0)	2 (25.0)
8–18 h and in the emergency room	–	1 (12.5)
Not available	2 (18.1)	–

In the eight non-referral centers, 24/7 continuity of service is available in four (50%) facilities (Friuli, Emilia Romagna, Lombardy, Sicily) (Table 3) and in two center (25%) the continuity of care is provided for 8–16 h (Table 3). Four centers (50%) have a dedicated team (two centers in Lombardy, Emilia Romagna and South Tyrol).

Nine centers approved a median of six beds (min–max: 2–10). The median of actually available beds is four (min–max: 0–10). In three out of nine centers (33%), fewer beds are available than approved.

### Activity in the period 2019–2021

In 2019, 2020, and 2021, 1,209, 1,202, and 1,544 patients were reported to be in the care of specialized PPC centers, respectively. Table 4 summarizes the activity data in from 2019 to 2021.

**Table 4:** Activity data in the period 2019–2021

	Number of centers	Mean (SD)	Min–max	Number of patients
**2019**				
Home care	15	49 (56)	0–220	729
Pediatric hospice	9	53 (101)	0–315	480
**2020**				
Home care	17	49 (57)	0–234	841
Pediatric hospice	10	36 (65)	0–216	361
**2021**				
Home care	18	55 (77)	0–332	993
Pediatric hospice	10	55 (101)	0–333	551

### Healthcare providers

Table 5 summarizes the distributions of healthcare providers among all participating centers and the related FTE. According to the FTE, most healthcare providers were nurses (*n* = 115 providers; 96 FTE), followed by physicians (*n* = 55; 43 FTE) and this distribution is observed in both referral and non-referral centers (Supplementary Table 1).

**Table 5:** Healthcare providers working in the participating centers

Healthcare providers	Total	FTE
Nurses	115	96
Physicians	55	43
Healthcare social workers	31	29
Psychologists	27	14
Physiotherapists	13	9
Other	18	11

FTE: full-time equivalent

Out of 115 nurses, 43% were over 46 years old (33% were over 55 years); 32% had more than 5 years of experience in PPC, and 18% had a master’s degree in PPC, while 77% had no training in PPC.

Out of 55 physicians, 66% were over 46 years old (33% were over 55 years); 43% had more than 5 years of experience in PPC, and 83% obtained a specialization according to legislation, mainly in pediatrics (60%); 54% had a master’s degree in PPC. In 17 (89%) centers, at least one physician had postgraduate training.

Out of 27 psychologists, 33% were over 46 years old (33% were over 55 years), 29% had more than 5 years of experience in PPC, and 30% had a master’s degree in PPC; 30% had no training in PPC.

No difference in these trends was observed between referral and non-referral centres (Supplementary Table 2; Supplementary Table 3; Supplementary Table 4).

## Discussion

PPC is essential for providing comprehensive, compassionate, and family-centered support for children with life-limiting and life-threatening conditions and their families, helping children to live as comfortably and fully as possible [1]. This includes managing pain, addressing emotional and spiritual needs, and promoting overall well-being throughout the course of the illness. Specialized PPC involves a multidisciplinary team of healthcare professionals working together to provide consistent and coordinated care [1]. This ensures that the child receives comprehensive and continuous support, regardless of changes in their condition. Accordingly, PPC is recognized as a right for children and their families [1, 6].

The complexity of the clinical situation of patients requiring specialized PPC pose real challenges in defining an organizational model able to meet the needs of these patients and their families. At the same time, continuous evolution parallels the ever-changing needs of patients in terms of treatment options, instruments, and supporting technologies. In this context, it is necessary to work on the definition of strategies and tools that allow patients in need of PPC and their families continuous preservation and improvement in their quality of life.

Up to date, few studies have addressed specialized PPC development and provision at the national level and the available data report an uneven availability of PPC services in Europe [10]. In particular, 19 out of 51 considered European countries, including Italy, reported specific standards and norms for the provision of PPC [10, 11]. In 48 of these 51 countries, 680 CPP provision units have been reported, divided into: 133 hospices in 21 countries; 385 Home Palliative Care Units in 29 countries; 162 In-hospital services in 27 countries; in seven countries, including Italy, at least one referral center for perinatal palliative care has been reported [11]. Overall, 22 countries had a national association, and 14 countries reported to offer education for either pediatricians or nurses [10]. PPC development was less accentuated in low- to middle-income countries [10]. Overall, these data suggest a global need for strategies to regulate and improve the provision of specialized PPC at a national level.

In the Italian scenario, the law 38/2010 promotes the creation of regional and national networks of palliative and pain care centers, coordinated by at least one regional referral center, to ensure the continuity of care from hospital to home through multidisciplinary clinical pathways (art. 2; art. 5) [8]. The Ministry of Health also promotes information campaigns involving all healthcare professionals and the population to spread public opinion and awareness of the relevance of PPC and pain management (art. 4) [8]. The above-mentioned law also supports educational and experimental programs that should implement the coordination and integration of the PPC services in the territory (art. 6) [8].

To enable the implementation of the organizational model promoted by the Ministry of Health, the assessment of the extent and quality of regional specialized PPC networks/facilities and the number of dedicated resources, as well as the characterization of this model of care, represent necessary steps for resource allocation by the NHS.

PalliPed is the first Italian nationwide project to collect and describe the characteristics of the Italian specialized PPC networks and facilities. Through a self-reported online survey, the activity of 19 PPC centers/facilities from 12 Italian regions and two autonomous provinces was described, providing the first comprehensive assessment of the extent and quality of PPC in Italy.

Among the 19 participating centers, 11 are regional referral centers. Seven Italian regions, mainly in central-southern Italy, reported no PPC centers/facilities. Overall, 1,092 patients were followed by 18 centers as of 24 October 2022.

Less than half (45%) of the regional referral centers cover the entire regional territory, and three offer 24/7 service. Ten centers have a dedicated team. Half of the eight non-referral centers offer 24/7 service and have a dedicated team.

Over the years, an increasing number of patients has been reported, rising from 1,202 (2019) to 1,544 (2021). According to previous estimates, the number of children requiring specialized PPC in Italy in 2021 was about 10,600 [5]. This suggests that, in the study period, the need for PPC was covered by only 15%. Therefore, despite the upward trend, the number of patients reported is far lower than the estimated number of children needing PPC. Causes of a low number of patients cared by PPC are diverse and intersecting. There is a basic cultural factor, for which PPCs are not an easy topic to discuss because they are assimilated to end-of-life care, to which is added the poor training of operators, who struggle to recognize the needs of patients and therefore to refer patients to referral centers. In addition, accessibility to the service is significantly limited, due to the limited number of dedicated centers and resources. This suggests that specialized PPC service in Italy is still scantly diffused, and requires prompt improvement by promoting a widespread and more organized system of care to cover the estimated need for PPC.

The resources in terms of dedicated staff are not adequate, and often, the team is only available on an as-needed or volunteer basis, with most healthcare providers not recognized at an institutional level. Moreover, a shortage of ‘young’ staff, with most healthcare providers over 46 years old, and a lack of specific training, particularly among nurses (77% with no training in PPC), emerged.

The data collected within this project emphasize the need for a greater effort to provide for the reorganization of care models and resources in PPC, to enable the proper implementation of the organizational model promoted by the Ministry of Health, since the number of children needing PPC is constantly increasing [12, 13]. In this context, the challenge for the future is the management of chronicity, especially for incurability and dependence on life-saving devices. At the same time, the evolving care environment within the PPC poses the problem of the economic sustainability of the proposed models and their networks [14]. While these allow quality and assistance to be ensured to PPC patients and their families, the lack of dedicated funding and support programs can lead to a slowdown in the realization and implementation of care pathways and networks, strongly impacting the actual possibility of assistance for these patients. Our study presents some limitations, such as the survey design and the self-reporting method of data collection. However, the PalliPed project establishes the first comprehensive database on the extent and quality of specialized PPC service in Italy, representing a fundamental step to support and facilitate the implementation and monitoring of the NHS support measures for PPC networks and facilities.

## Conclusion

The PalliPed project provides the first comprehensive database on the characteristics of Italian patients requiring specialized PPC and their families and the extent and quality of the PPC service in Italy. The results show how training, information, and research interventions are still necessary for the reorganization of the available resources and for the definition of proper strategies to respond dynamically to the new emerging needs of these patients, according to the model of care promoted by the Ministry of Health. At the same time, our study is a first step toward establishing a national registry of patients and PPC models, useful for monitoring evolutions and critical issues and to plan and implement any new or corrective strategy. In addition, other settings in different countries can apply this research model, allowing comparison of different PPC care models.

### Electronic supplementary material

Below is the link to the electronic supplementary material.

Supplementary Material 1

Supplementary Material 2

## Data Availability

All data generated or analyzed in this study are included in this article and/or its figures. Further inquiries can be directed to the corresponding author.

## Abbreviations

PPC: Pediatric palliative care
FTE: Full-time equivalent
PT: Pain therapy
