# Supplementary material for: The specialized pediatric palliative care service in Italy: how is it working? Results of the nationwide PalliPed study
Source: Ital J Pediatr. 2024 Mar 19;50:55. doi: 10.1186/s13052-024-01604-1 (PMC10953081; doi:10.1186/s13052-024-01604-1)
Supplement: Supplementary file 1 — Supplementary Material 1 [file 13052_2024_1604_MOESM1_ESM.docx]

**Questionnaire on PPC centers/facilities**

* **Specify your region/autonomous province** (drop-down menu)

#### * ****Type of service** (multiple answers)**

- Regional pain therapy and pediatric palliative care network
- Regional referral center for pediatric palliative care and pain therapy
- Pediatric palliative care residential center (pediatric hospice)
- Specialized pediatric palliative care team
- Pediatric palliative care facility or service

#### *  ****Name of network/regional referral center/facility/service****

*  **Facility contact information** w

*Phone*

*Address*

*Email*

*  **Respondent** w

*First name*

*Last name*

*Profession*

*Phone*

*Email*

#### *  ****Date of completion****

#### *  ****The network/regional referral center/pediatric hospice/pediatric palliative care specialty team is:****

- Public entity
- Accredited private entity
- Other (specify)

#### * ****Is the entity you are presenting a regional referral center for PPC and PT?****

- Yes
- No

#### ****If not, does it collaborate with the regional referral center for PPC and PT?**** w

- Yes
- No
- There is no deliberate referral center for PT and PPC in our region

#### * ****What are the care settings in which the presenting institution operates?**** w

- Pediatric hospice
- Home
- Hospital, outpatient
- Hospital, inpatient
- Other (specify)

#### * ****Is home care continuity activated on taking care for a 24-hour time slot, 7 days a week?**** w

- Yes
- No

#### * ****If no, specify the time slot for home care continuity****

#### * ****Territorial coverage**** w

- It covers the entire regional territory (or provincial, for autonomous provinces)
- It covers only some areas of the regional territory (specify which ones)

#### * Medical team w

- The activity is carried out by a specialized multidisciplinary team of dedicated and structured healthcare providers (i.e., belonging to simple or complex facilities) who follow patients in different care settings (home, pediatric hospice, hospital)
- The activity is carried out only by a small specialized multidisciplinary team of dedicated and structured healthcare providers (i.e., afferent to simple or complex structures), also supported by healthcare providers working part-time in other organizational structures (adult palliative care) who intervene in the management of eligible subjects.
- The activity is carried out by non-dedicated professionals who also work in different facilities and who intervene in the management of eligible patients in non-institutionalized teams
- Other (specify)

#### * ****Number of pediatric hospice beds deliberated****(Indicate a whole number; indicate NA if no pediatric hospice is present)

#### *  ****Number of pediatric hospice beds activated**** (Indicate a whole number; indicate NA if no pediatric hospice is present)

#### * ****Please indicate the activity data, 2019**** (indicate NA if not applicable) w

- Number of patients assisted at home in 2019
- Number of patients admitted to PH in 2019

#### * ****Activity data, 2020****(indicate NA if not applicable) w

- Number of patients assisted at home in 2020
- Number of patients admitted to PH in 2020

#### * ****Activity data, 2021****(indicate NA if not applicable) w

- Number of patients assisted at home in 2021
- Number of patients admitted to PH in 2021

#### * ****Healthcare personnel working in the network/regional referral center/pediatric hospice/pediatric palliative care specialist teams**** (Indicate the number of the healthcare providers. If they are not present, indicate 0) w

Physicians

Nurses

Psychologists

Physiotherapists

Healthcare social workers

Other healthcare providers

#### *  ****FTE of dedicated operating healthcare personnel****(Indicate the FTE number. If they are not present, indicate 0) FTE (Full Time Equivalent) equals one person working full time (regardless of the type of employment contract) (e.g., two 50% part-timers equal one total unit) w

Physicians

Nurses

Psychologists

Physiotherapists

Healthcare social workers

Other healthcare providers

#### *  ****For each physician, indicate the following information****

#### *Age*

#### *Experience in PPC (years)*

#### *Specialty*

#### *Postgraduate training*

#### *  ****For each healthcare provider** (specify which one), **please indicate the following information****

#### *Age*

#### *Experience in PPC (years)*

#### *Postgraduate training*

#### ****If other personnel are present, indicate their duties and briefly describe their training and experience.****

####

#### *  ****Can other specialized skills be activated based on specific needs/cases?****

- Yes
- No

**If yes, which ones?**
