# Supplementary material for: The specialized pediatric palliative care service in Italy: how is it working? Results of the nationwide PalliPed study
Source: Ital J Pediatr. 2024 Mar 19;50:55. doi: 10.1186/s13052-024-01604-1 (PMC10953081; doi:10.1186/s13052-024-01604-1)
Supplement: Supplementary file 2 — Supplementary Material 2 [file 13052_2024_1604_MOESM2_ESM.docx]

**The pediatric palliative care service in Italy: how is it working? Results of the nationwide PalliPed study**

**SUPPLEMENTARY MATERIAL**

**Glossary**

| **Regional Network of Pain Therapy and Paediatric Palliative Care** |
| --- |
| Regional Network of Pain Therapy (PT) and Paediatric Palliative Care (PPC) means a functional and integrated aggregation of pain therapy and pediatric palliative care activities, provided in the different settings of care, in a territorial and hospital area defined at regional level (State-Regions Agreement of 25 July 2012, Rep. acts n. l5l / CSR).  The organizational and functional requirements meet the criteria of the Accreditation Document of the SR Conference of 23 March 2021 |

| **Regional Referral Centre for Paediatric Palliative Care and Pain Therapy** |
| --- |
| Regional Referral Centre for Paediatric Palliative Care (PPC) and Pain Therapy (PT) means the clinical, organizational, training and research reference structure of the Network. The Center is a public structure, established by a formal act of the Region, dedicated and specific from an organizational point of view and resources for the pediatric age, with its manager.  This structure requires the presence of a dedicated, specialized team that, with different skills, works in a multidisciplinary way to respond to the multiple needs of children and families, both at hospital level and at home, in continuity of care, objectives and choices. The Centre guarantees continuity of care by offering availability 24/7.  The organizational and functional aspects must meet the criteria of the Agreement of 25 July 2012 (Rep. Acts no. I 5 I/CSR) and the Accreditation Document of the SR Conference of 23 March 2021. |

| **Paediatric Palliative Care Residential Centre (Paediatric Hospice)** |
| --- |
| Pediatric Hospice means an alternative residential structure to the hospital, dedicated and specific only to the pediatric patient and possibly to the young adult. It is a structure inserted within the regional network of PT and PPC, with high care complexity, independent and autonomous from a logistic, organizational and managerial point of view.  The organizational and functional requirements meet the criteria of the SR Conference Accreditation Document of 23 March 2021. |

| **Specialist Paediatric Palliative Care Team (not Centre or Regional Network)** |
| --- |
| The specialist PPC team is made up of dedicated professionals with proven experience in PPC and pain therapy, as well as in highly complex specialist activities. The team guarantees the specialized management of PPCs in interaction and integration with the disease specialist and all the other actors of the Network, providing hospital and home services.  The organizational and functional requirements meet the criteria of the SR Conference Accreditation Document of 23 March 2021. |

| **Paediatric palliative care facility or service** |
| --- |
| Team dedicated to palliative care, integrated within other services (e.g., Paediatrics or Anaesthesia) |

**Supplementary Tables**

| **Supplementary Table 1. FTE of healthcare providers working in the participating centers** | | |
| --- | --- | --- |
| **Healthcare providers** | **Referral centers (N=11)** | **Non-referral centers**  **(N=8)** |
| Physicians  Median (min–max)  Physician/Patients Ratio | 2 (1–7)  0.04 (0.01–1.2) | 1 (0–4)  0.03 (0–0.3) |
| Nurses  Median (min–max)  Nurse/patient ratio | 4 (0–24)  0.07 (0–2.2) | 2 (0–13)  0.05 (0–0.7) |
| Psychologists  Median (min–max)  Psychologists/patients ratio | 0.2 (0–2.8)  0.003 (0–0.3) | 0.3 (0–2)  0.006 (0–0.3) |
| Physiotherapists  Median (min–max)  Physiotherapists/patients ratio | 0 (0–2)  0 (0–0.2) | 0 (0–2)  0 (0–0.3) |
| Healthcare social workers  Median (min–max)  HSW/patients ratio | 0 (0–7)  0 (0–0.8) | 0 (0–6)  0 (0–0.7) |
| Other operators  Median (min–max)  Other operators/patients ratio | 0 (0–1)  0 (0–0.2) | 0 (0–5)  0 (0–0.1) |

| **Supplementary Table 2.** Nurses features according to referral or non-referral centers. | | |
| --- | --- | --- |
|  | **Referral centers** | **Non-referral centers** |
| **Age (years)** |  |  |
| 18–24 | 3 (4%) | – |
| 25–35 | 14 (21%) | 13 (30%) |
| 36–45 | 20 (29%) | 13 (30%) |
| 46–55 | 22 (32%) | 11 (26%) |
| >55 | 9 (13%) | 6 (14%) |
| Total | 68 (100%) | 43 (100%) |
| **Experience in PPC (years)** | | |
| 0–1 | 11 (16%) | 11 (26%) |
| 2–5 | 38 (56%) | 19 (44%) |
| 6–10 | 7 (10%) | 12 (28%) |
| >10 | 12 (18%) | 1 (2%) |
| **Postgraduate training** | | |
| Master PC | 3 (4%) | 6 (14%) |
| Master PPC | 12 (18%) | 6 (14%) |
| None | 53 (78%) | 31 (72%) |

| **Supplementary Table 3.** Physicians features according to referral or non-referral centers. | | |
| --- | --- | --- |
|  | **Referral centers** | **Non-referral centers** |
| **Age (years)** |  |  |
| 25–35 | 2 (6%) | - |
| 36–45 | 8 (24%) | 8 (40%) |
| 46–55 | 10 (29%) | 8 (40%) |
| >55 | 14 (41%) | 4 (20%) |
| Total | 34 (100%) | 20 (100%) |
| **Experience in PPC (years)** | | |
| 0–1 | 1 (3%) | - |
| 2–5 | 19 (56%) | 11 (55%) |
| 6–10 | 6 (18%) | 8 (40%) |
| >10 | 8 (24%) | 1 (5%) |
| **Specialization according to legislation** | | |
| Anaesthesia | 10 (29%) | 2 (10%) |
| Paediatrics | 19 (56%) | 14 (70%) |
| Other Specialty | 3 (9%) | 4 (20%) |
| Not known | 2 (6%) | – |
| **Postgraduate training** | | |
| Master PC | 1 (3%) | 1 (5%) |
| Master PPC | 19 (56%) | 10 (50%) |
| Other | 6 (18%) | 2 (10%) |
| None | 8 (23%) | 7 (35%) |

| **Supplementary Table 4.** Psychologists features according to referral or non-referral centers. | | |
| --- | --- | --- |
|  | **Referral centers** | **Non-referral centers** |
| **Age (years)** |  |  |
| 18–24 | 1 (6%) | - |
| 25–35 | 2 (13%) | 2 (18%) |
| 36–45 | 8 (50%) | 5 (46%) |
| 46–55 | 4 (25%) | 4 (36%) |
| >55 | 1 (6%) | - |
| Total | 16 (100%) | 11 (100%) |
| **Experience in PPC (years)** | | |
| 0–1 | 5 (31%) | 1 (9%) |
| 2–5 | 7 (44%) | 6 (55%) |
| 6–10 | 2 (13%) | 2 (18%) |
| >10 | 2 (13%) | 2 (18%) |
| **Postgraduate training** | | |
| Master PC | 2 (13%) | 1 (9%) |
| Master PPC | 6 (38%) | 2 (18%) |
| Other | 4 (25%) | 2 (18%) |
| None | 4 (25%) | 6 (55%) |
